# Prevalence of Transfusion Transmissible Infections in Beta-Thalassemia Major Patients in Pakistan: A Systematic Review

**DOI:** 10.7759/cureus.10070

**Published:** 2020-08-27

**Authors:** Hamid Ehsan, Ahsan Wahab, Faiz Anwer, Raheel Iftikhar, Muhammad N Yousaf

**Affiliations:** 1 Biomedical Sciences/Biohazardous Threat Agents & Emerging Infectious Diseases, Georgetown University, Washington, USA; 2 Internal Medicine, MedStar Union Memorial Hospital, Baltimore, USA; 3 Internal Medicine, Baptist Medical Center South, Montgomery, USA; 4 Hematology and Oncology, Cleveland Clinic, Cleveland, USA; 5 Hematology and Oncology, Armed Force Bone Marrow Transplant Center/National Institute of Blood and Marrow Transplant, Rawalpindi, PAK; 6 Internal Medicine, MedStar Franklin Square Medical Center, Baltimore, USA; 7 Internal Medicine, MedStar Good Samaritan Hospital, Baltimore, USA; 8 Section of Digestive Diseases, Yale University School of Medicine, New Haven, USA

**Keywords:** thalassemia major, beta thalassemia major, pakistan, prevalence, transfusion transmissible infections, hepatitis b, hepatitis c, hiv

## Abstract

*β*-thalassemia major (TM) is one of the most prevalent inherited hemoglobinopathies in Pakistan. It has one of the highest prevalence of transfusion-dependent TM patients globally, with an estimated greater than 100,000 active cases. Blood transfusions (BT) are essential in the management of severe TM; it is critical to have a safe BT to reduce the risk of transfusion transmissible infections (TTIs). Frequent blood transfusions in these patients increase their risk of acquiring TTIs compared to the general population. We performed a systematic literature search to identify studies related to the TTIs and transfusion-related infections in Pakistan from January 1, 2010, to January 31, 2020. The search was conducted using PubMed and PakMediNet, with initial search retrieved 981 studies. Among these, 166 studies met the inclusion criteria, and only 14 studies met the final criteria for qualitative synthesis. Analysis of 14 studies (n = 3786) showed the seroprevalence of hepatitis B virus (HBV) of 3.13% (0.66% to 7.4%) and hepatitis C virus (HCV) of 26% (5.56% to 68.2%). There were only two studies that reported HIV seroprevalence of 0% and 0.5% (n = 6). The rate of seropositivity for HBV and HCV was directly related to the number of transfusions, higher ferritin levels, and older age groups. There was an increase in the HCV rate with the increasing age of patients. Thalassemia patients, who were older than ten years of age, had an HCV rate of 22% compared to only 8.4% in patients younger than ten years of age. A comparison of HCV in healthy donors vs. thalassemia patients showed a rate of 1.9% vs. 13.1% for TM patients. The majority of the patients were males (51% to 88%). The seroprevalence of TTIs was higher in males than in females (73.4% vs. 26.6%). On average, a single TM patient is exposed to at least 17 different donors annually, requiring 1-2 transfusions every month. Our study highlights that the prevalence of transfusion-transmitted infections, especially HCV, is alarmingly higher (26%) in the TM population than in the general population. There is limited data regarding the prevalence of HIV, syphilis, and malaria in this population. This is mainly due to a fragmented system of blood transfusion, weak regulations, and lower rates of voluntary blood donations. These findings warrant better health measures to improve the blood donation system and specialized care for TM patients.

## Introduction and background

*β*-thalassemia major (TM) is one of the most common inherited hemoglobinopathies in Pakistan, with a gene carrier rate of 5-7% and roughly a pool of 9.8 million carriers in the general population [1]. Currently, approximately 50,000 thalassemia patients are registered with the treatment centers throughout the country [2]. It is estimated that Pakistan has one of the highest prevalence of transfusion-dependent TM patients in the world [3]. This is mainly due to a high frequency of hemoglobin β-subunit (HBB) gene mutation, high birth rate, larger population size, and traditional practices of consanguineous marriages [4]. Every year, an estimated 5000-9000 new cases of TM are being diagnosed in the country [5, 6]. Historically, β-thalassemia had been associated with high mortality and morbidity. However, there is a remarkable improvement in its global outcomes and a significant decrease in all-cause mortality [7-9]. There are substantial clinical and scientific advancements in the management of TM care, including evidence-based safe blood transfusion (BT) practices, availability of iron chelation therapy, bone marrow transplantation, innovative gene therapy and a reduction in transfusion transmissible infections (TTIs) [10, 11]. TTIs are the leading causes of morbidity and mortality in TM [8, 11]. Since BTs are essential in the management of severe thalassemia, it is critical to have a safe BT to reduce the risk of TTIs. The average life expectancy of TM patients has improved closer to the 6th decade in the Western world due to better treatment options and public health measures. However, in Pakistan, it still averages around ten years of age [12, 13]. This disparity exists due to a lack of organized and adequately equipped transfusion services, which results in a higher risk of TTIs, iron overload, hepatoxicity, and cardiac complications. TTIs are the second leading cause of death in TM patients in Pakistan, and TTIs prevalence is significantly higher in TM patients as compared to the general population (29.3% vs. 7.2%) [14].

Hepatitis C virus (HCV) infection is the most prevalent TTI in thalassemia following hepatitis B virus (HBV) infection, and an increased risk of contracting human immunodeficiency virus (HIV) [14, 15]. The rate of infections in TM patients may serve as a marker for the high prevalence of TTIs in the general population. Several studies have reported a variable rate of TTIs among TM patients in Pakistan, and the exact prevalence is still undetermined [16-19]. The purpose of this systematic review is to analyze the available data and evaluate the overall prevalence of TTIs in TM patients chronically dependent on blood transfusions.

## Review

Materials and method

**Figure 1:**
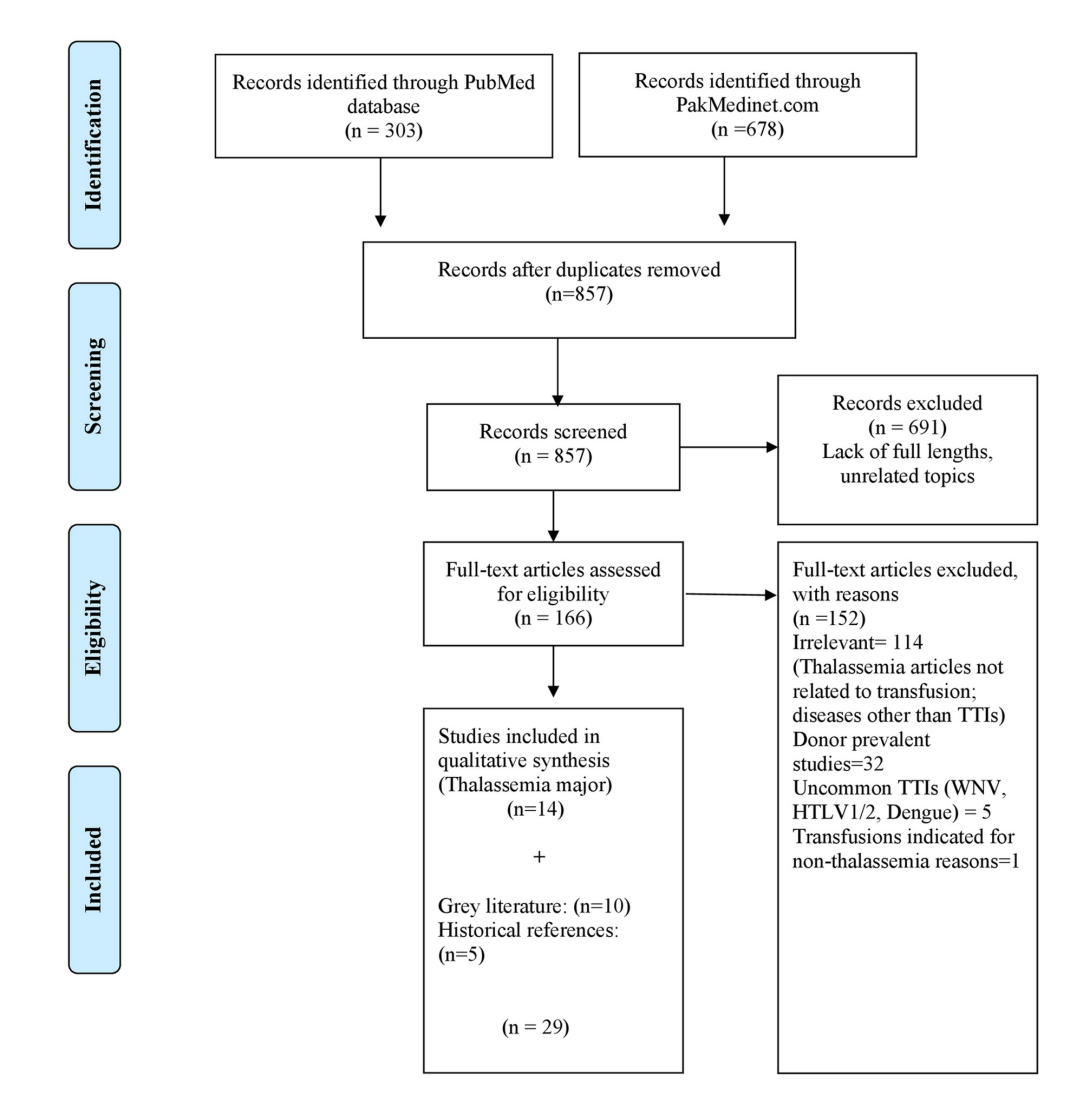
Flowchart summary of the selection process.

We conducted a systematic literature search on PubMed and PakMediNet (Pakistan’s largest medical database) for studies on TTIs in Pakistan that are published between January 1, 2010, and January 31, 2020. We searched for broader terms such as transfusion-transmitted infections, transfusion-related infections, and blood transfusion infections. Studies published in the languages other than English, outside of the timeframe, metanalysis, and systematic reviews were excluded. Studies without a full-text draft were also excluded. Studies with a prevalence of TTIs in chronically transfused TM recipients were included. Initial PubMed and PakMediNet search retrieved 981 studies. After removing the duplicates, we reviewed 857 studies, out of which 166 relevant studies with full available text were narrowed down. We excluded 152 studies with reasons, as indicated in the PRISMA flow chart (Figure 1). Only 14 studies on TTIs in thalassemia major were analyzed in this systematic review.

Results

We analyzed 14 studies with a total of 3786 thalassemia patients on the prevalence of TTIs in chronically transfusion-dependent TM patients (Table 1). A cumulative seroprevalence of individual TTIs was calculated by dividing the number of positive results for HBV, HCV, and HIV, with a total number of TM patients.

**Table 1:** Studies on the prevalence of transfusion transmissible infections in chronically transfusion dependent thalassemia patients. HBV: hepatitis B virus; HCV: hepatitis C virus; n: number of patients; NR: not reported. * The positive results calculated prevalence in each study for HBV, HCV, or HIV by any method divided by the total number of thalassemia patients who were tested for that transfusion-related infection.

Author	Year	Number of patients, n	Prevalence* of HBV n (%)	Prevalence of HCV n (%)	Prevalence of HIV n (%)
Rehman et al. [16]	2011	150	NR	45 (30%)	NR
Ansari et al. [17]	2012	160	2 (1.25%)	21 (13.1%)	0 (0%)
Shahid et al. [19]	2013	150	1 (0.66%)	77 (51.3%)	NR
Akram et al. [20]	2013	58	NR	22 (37.9%)	NR
Nazir et al. [21]	2014	200	NR	82 (41%)	
Sheikh et al. [22]	2015	145	5 (3.5%)	99 (68.2%)	NR
Ali et al. [23]	2016	379	NR	123 (32.45%)	NR
Kiani et al. [24]	2016	1253	38 (3%)	273 (21.7%) HBV/HCV coinfection: 2 (0.16%)	6 (0.5%)
Sultan et al. [25]	2016	100	3 (3%)	27 (27%)	NR
Rashid et al. [26]	2017	130	8 (6.2%)	27 (20.8%)	NR
Shah et al. [27]	2019	324	6 (1.85%)	18 (5.56%)	NR
Jehan et al. [28]	2019	292	3 (1%)	53 (18.2%) HBV/HCV coinfection: 1%	NR
Abid et al. [29]	2019	95	2 (2.1%)	6 (6.31%)	NR
Yasmeen et al. [30]	2019	350	26 (7.4%)	103 (29.4%) HBV/HCV coinfection: 21 (6%)	NR
Total prevalence		3786	94/2999 = 3.13%	976/3786 = 26%	NR

The prevalence of male gender was predominant in the study population and ranged from 51% to 88%. The seroprevalence of TTIs was higher among male vs. female (73.4% vs. 26.6%), patients older than ten years, and those with siblings suffering from thalassemia. On average, a single TM patient is exposed to at least 17 different donors annually, requiring 1-2 transfusions every month. The free BT is accessible only in one out of four thalassemia centers, and the majority of patients either need to bring their donors or they are dependent on an external source of financial aid as they could not afford the cost of BT treatment. More than half of thalassemia patients (57.2%) need to contact multiple BT centers in the search for required blood products. About 42.1% of parents of TM patients did not know about TTIs, whereas 31.6% of them did not know about the bloodborne transmission of HBV and HCV. The majority of parents of TM children had a low income, with 75% of them having income less than 10,000 Pakistani rupees (PKR) per month.

Although reported HBV seroprevalence ranged from 0.66% to 7.4%; however, in our analysis, a cumulative seroprevalence of HBV was 3.13% (n = 94). Factors associated with higher rates of seroprevalence in some studies are male gender, age older than ten years, unvaccinated status for HBV, and increasing number of blood transfusions (>20 transfusions). The overall low HBV seroprevalence as compared to HCV in TM patients were thought to be due to a remarkably high prevalence of HBV vaccination rate in these patients (79.8%).

The reported seroprevalence of anti-HCV antibodies ranged from 5.56% to 68.2%; however, in our analysis, a cumulative seroprevalence of anti-HCV antibodies was 26% (n = 976). Factors associated with higher rates of anti-HCV antibodies highlighted in studies include a number of BTs, age of patients, male gender, and serum ferritin level. A higher anti-HCV antibody was noted in males (23.6%) vs. females (19.6%). The group with more than 50 BTs had 93% reactive cases as compared to 7% in a group of fewer than 50 BTs. Anti-HCV was not reactive in patients with less than 20 BTs. There was 74% of HCV infection in the group with greater than 100 BTs compared to 33% in a group with fewer than 35 BTs. The rate of HCV increased to 75% for the patients who had more than 100 BTs. The mean number of BTs was more significant for HCV reactive cases vs. non-reactive cases.

There was an increase in the HCV rate with the increasing age of patients. Thalassemia patients who were older than ten years of age had a greater HCV compared to those who were less than ten years of age, i.e., 22% vs. 8.4%, p: 0.005, respectively. The mean age was higher in HCV reactive children than non-reactive children. A comparison of HCV in healthy donors vs. thalassemia patients showed a rate of 1.9% vs. 13.1% for TM patients. HCV infection rate was 96% in thalassemia children as compared to 4% in other disorders requiring multiple transfusions such as leukemia, aplastic anemia, and thrombasthenia, etc.

There were only two studies that estimated the rate of HIV in TM. There was no HIV reactivity noted by ELISA in 160 subjects (n = 0). And in other study rates of 0.5% (n = 6) were reported. There was a rate of 0.68% (n = 26) having coinfection with both HBV and HCV.

Discussion

Pakistan has the second-highest number of HCV cases in the world (>10 million), with a prevalence of 5.9% in the general population [31]. Our results showed a seroprevalence of 26%, though, a variable range of HCV prevalence has been reported in prior studies (5.5% to 68.2%) [19, 22, 26-30]. Current prevalence of HCV in TM patients is 13.6% in Iran, 14.7% in Bangladesh and 11-30% in India [32-34]. This significant prevalence of HCV in Pakistan is predominantly due to a lack of centralized system, specialized care centers, voluntary remunerated blood donations (VNRDs), low socioeconomic status of patients, and improper blood donor screening [35]. The majority of these patients have limited access to regular and safe blood transfusions [30]. Only 12% of patients receive a standardized transfusion, while 40% have access to iron chelation therapy [36]. Our results showed HBV is the second most common TTI in TM patients with a prevalence of 3.13%, while its reported seroprevalence ranged from 0.66% to 7.4% [19, 22, 26-30]. Our findings were similar to the majority of studies [19, 22, 26-30], but other studies reported variable results due to variations in the disease prevalence in various regions of the world and non-standardized methods of transfusion in some centers due to lack of facilities. The prevalence of HBV is found to be lower than HCV is likely due to higher rates of immunization against HBV. HBV vaccination is highly effective (80-100%) in reducing the rate of HBV infections in those who receive the complete vaccine series [37]. Although the prevalence of HBV is lower than HCV due to improved vaccination status, however, it is still on the higher side as compared to the global prevalence of HBV in thalassemia patients, which ranges from 0.3% to 5.7% [38]. HBV vaccination status is improving, though not optimized given only 27.5% of thalassemia patients and families were aware of the need for vaccination due to lack of knowledge, resources, economic hardships, and proper counseling [6].

The prevalence of HIV in blood donors is less than 0.18% [39]. The alarming trend noticed in our analysis is that only two studies reported HIV with a prevalence of 0.5% in TM patients, which is much higher than the prevalence of HIV in the donor population. This indicated that TM patients are not widely tested and monitored for HIV. There is an urgent need for regular HIV testing to better assess the risk and actual burden of the disease in the thalassemia population.

The serious challenges contributing to the high prevalence of TTIs in TM patients in Pakistan are lack of knowledge, low socioeconomic status, limited access to the safe B.T. practices, use of family-directed replacement donors (RDs) rather than VNRDs, and unavailability of specialized thalassemia centers for the majority of patients [30]. In a study assessing the awareness of TTIs, only 15.8% of patients and families knew the importance of adequate blood screening, only 15% knew about the risk of family inheritance, and only 5.8% had family screening [6]. The affected population is mostly unaware of the importance of blood screening, VNRDs, and regular testing for TTIs. Moreover, nucleic acid amplification test (NAT) screening is available at very few large thalassemia centers, and the majority of other centers rely on ELISA and even rapid testing kits for screening [23]. This poses a serious risk of missing out on TTIs. Red cell phenotyping and pre-storage leucodepletion are not done by all the thalassemia centers in Pakistan, resulting in alloimmunization and increases transfusion requirements, which further increases the risk of TTIs [40]. A high cost of standardized care of TM ($4500 per year) is an additional factor contributing to a higher rate of TTIs in Pakistan where the average annual income of individual families is less than $587 with more than 35% of the population living below the poverty line [18, 41]. The majority of TM patients cannot afford the cost of regular BTs and have limited access to thalassemia centers [30]. In our review, the majority of TM patient families had a low income with more than 75% of them earning less than $150 (~25,000 PKR) a month and could not afford the cost of BT treatment without significant external funding from private organizations, public donations, and NGOs (non-governmental organizations) [30].

Currently, there is a need for specialized thalassemia centers for care and awareness of TM disease in patients and their families. These measures have demonstrated a positive impact on the inheritance, prevention, and management of the disease in the affected population with specialized center focused care [42]. Also, for better evaluation of TTIs in the future, there is a need for establishing the thalassemia registry for uniform data gathering, development of a standard care system, monitoring, and improving the quality of thalassemia care. Large prospective multi-centered clinical trials are required for a better understanding of the high prevalence of TTIs in patients with TM.

This review has limitations. As it is based on the data from studies that focused on localized geographical locations due to the lack of availability of large scale-multicenter structured data. Therefore, it has limited generalizability. We relied on data sources to find the most recent and consistent information that may lead to inherent reporting bias for the studies and their ability to get published. The included studies are of variable quality and scope. There is a risk of missing or omitting some publications due to reviewer bias. Moreover, the use of grey literature such as WHO and local reports are also based on limited data and relies on information reported by the government agencies. We recommend large scale prospective multicentered studies including the rural centers in Pakistan and stress to incorporate uniform well-planned safety measures in the care of thalassemia patients.

## Conclusions

Our study corroborates the findings of previously published data on the high prevalence of TTI, especially HCV (26%) in the TM population, as compared to the general population in Pakistan. The possible reasons include insufficient resources, inadequate safety measures, and a fragmented blood transfusion system. In short, we suggest the implementation of robust national and regional level policies regarding safe blood transfusion practices, VNRD-based transfusions, and universal quality-assured donor screening to minimize the future risk of TTI. Without these positive interventions, the current system of transfusion can lead to a further worsening of the situation.
